# Use of Large Language Models to Enhance Failure Mode and Effects Analysis: A Case Study

**DOI:** 10.1016/j.adro.2026.102060

**Published:** 2026-04-18

**Authors:** Saurabh S. Nair, Laurence Court, Raphael Douglas, Skylar Gay, Pavel Govyadinov, Tucker Netherton, Dong Joo Rhee, Josiane Pafeng, Lifei Zhang, Callistus Nguyen, Christoph Trauernicht, Christoffel van Reenen, Henriette Burger, Kailin Naidoo, Andrea Marais, Karl Sachse, William Shaw, Alicia Sherriff, Yao Zhao

**Affiliations:** a Department of Radiation Physics, The University of Texas MD Anderson Cancer Center, Houston, Texas, USA; b Division of Medical Physics, Tygerberg Hospital, Stellenbosch University, Cape Town, South Africa; c Department of Medical Physics, University of the Free State School of Medicine, Bloemfontein, South Africa

## Abstract

**Purpose:**

Failure Mode and Effects Analysis (FMEA) is widely used in radiation oncology to proactively identify and mitigate risks, but it is time-consuming and depends heavily on expert experience. This study evaluated whether large language models (LLMs) can supplement traditional expert-driven FMEA by identifying novel failure modes within the Radiation Planning Assistant (RPA) workflow.

**Methods and Materials:**

A multidisciplinary team of board-certified medical physicists, quality assurance engineers, and software developers independently used 4 LLMs (ChatGPT-4, Gemini 2.5 Pro, phi4-reasoning-14B, and OpenAI/oss-120B) to generate potential failure modes across the RPA contouring and planning workflow. Team members used diverse prompting strategies, including supplementary materials such as RPA user guides, as context. Each failure mode was first rated for severity, occurrence, and detectability by the LLMs, then independently rescored by experts using the TG-100 framework to enable comparison. The highest-risk modes, based on expert scoring, were subsequently reviewed with 2 clinical user groups in South Africa.

**Results:**

The 4 LLMs collectively generated 190 candidate failure modes. After review for relevance and duplication, 79 unique and interpretable modes were retained for analysis. Among these, 3 exceeded the 125 risk priority number threshold from a prior study, all related to staff accountability and role ambiguity. On average, LLMs assigned higher severity (7.3 vs 4.1), similar occurrence (2.8 vs 3.3), and lower detectability (5.4 vs 2.8) scores, producing higher mean RPNs (110 vs 36). Clinical users from 2 centers in South Africa confirmed that several artificial intelligence-identified risks were plausible, particularly those tied to workflow accountability.

**Conclusions:**

LLMs can broaden risk discovery in FMEA by surfacing contextually relevant and previously unrecognized failure modes. However, expert oversight remains essential for validating and prioritizing risks. Artificial intelligence should be viewed as a complementary tool that enhances, rather than replaces, human judgment in radiation therapy safety assessments.

## Introduction

In 2022, there were nearly 20 million new cancer cases worldwide, underscoring the continued rise in the global cancer burden.^1^ Radiation therapy offers a cost-effective means of cure and palliation, serving about 50% of all cancer patients, and as many as 60% to 70% in low-income settings, where late-stage disease at presentation is more prevalent.^2^^,^^3^ In low- and middle-income countries, cancer centers struggle to keep pace with the increasing complexity of radiation therapy, and only 37% of patients are estimated to have access to the necessary treatment.^4^ The adoption of artificial intelligence (AI)-based automation in radiation oncology, particularly in treatment planning, is accelerating as a strategy to address the increasing disease burden and limited access to care.^5^, ^6^, ^7^ The Radiation Planning Assistant (RPA), developed at MD Anderson Cancer Center, is a web-based platform that fully automates contouring and treatment planning, aiming to reduce time, resource requirements, and workforce barriers.^8^^,^^9^

The development and clinical implementation of new tools should be accompanied by a full risk management strategy. Failure Mode and Effects Analysis (FMEA) is a proactive framework for mapping complex processes, identifying potential points of failure, and prioritizing safeguards by scoring each failure mode on severity, occurrence, and detectability.^10^, ^11^, ^12^, ^13^, ^14^ In radiation oncology, FMEA is recommended by the AAPM TG-100 report and has been applied across planning, imaging, and delivery to strengthen patient safety.^15^ An initial FMEA study of the automated 4-field box plan for cervical cancer in the RPA identified 68 potential failure modes and showed that a specialized quality assurance (QA) program reduced risk priority numbers.^12^ A subsequent analysis of automated contouring and planning, again for the RPA, revealed 290 possible failure modes, 126 unique to automation, and demonstrated that user interface simplifications and enhanced training lowered mean and maximum risk priority numbers (RPNs).^14^ Performing such detailed FMEAs, however, requires substantial time and resources from multidisciplinary teams.^16^

Recent advances in large language models (LLMs) provide a new avenue for supporting risk assessment. LLMs are AI systems trained on vast collections of text that can generate structured outputs, synthesize knowledge, and propose contextually relevant ideas. In health care, LLMs have shown potential across diverse applications, including clinical documentation support and error detection.^17^, ^18^, ^19^, ^20^, ^21^, ^22^, ^23^, ^24^ Applied within a structured framework like FMEA, LLMs could complement expert-driven analyses by broadening the range of potential vulnerabilities considered. By suggesting failure modes that might not emerge during human brainstorming, LLMs may help uncover overlooked risks and strengthen the robustness of risk analysis.^25^ The objective of this study was therefore to evaluate whether LLMs could generate new and plausible failure modes within the RPA workflow that were not captured in earlier expert-driven analyses.

## Methods and Materials

### RPA workflow

**Figure 1:**
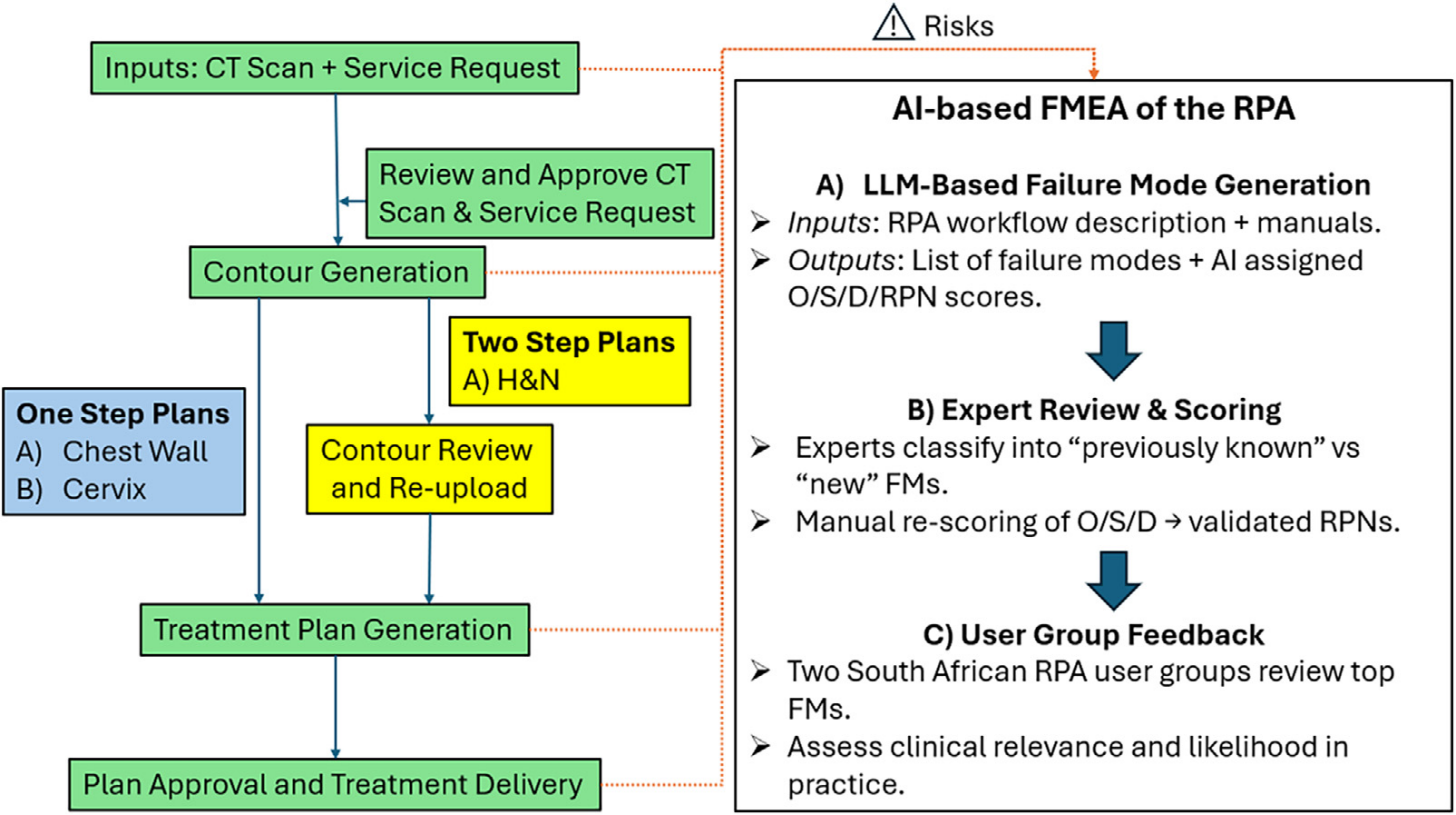
RPA workflow with integrated artificial intelligence (AI)-based Failure Mode and Effects Analysis (FMEA). Large language models (LLMs) were used to generate candidate failure modes and assign preliminary risk priority number (RPN) scores. These were then manually reviewed, rescored by experts, and subsequently shared with clinical user groups for external feedback. *Abbreviations:* CT = computed tomography; D = detectability; FMs = failure modes; H&N = Head and Neck; RPA = Radiation Planning Assistant; S = severity.

The overall RPA workflow, depicted in Fig. 1, was previously described by Court et al^8^ where the user interacts with the RPA via a web-based interface. The user needs to provide the RPA with 2 inputs, an approved computed tomography (CT) scan and an approved plan order. A user (eg, therapist) must approve the CT, and a radiation oncologist must approve the plan order, which contains patient information such as prescription and treatment site. Once both of them are approved, the treatment plan is automatically generated. For certain treatment types, the subsequent steps are fully automated whereas for some, manual intervention is required midway, with the user reviewing the contours generated before entering the treatment planning phase. Finally, once the planning phase is complete, the RPA outputs the generated plan as a PDF for review. The user then downloads the treatment files for review in their own treatment planning system, recalculation of the radiation dose, and routine QA tasks before proceeding with the treatment.

### AI-assisted FMEA of RPA

For this study, we assembled a multidisciplinary group that included 3 board-certified medical physicists with a strong understanding of patient treatment and safety, and 3 software developers with expertise in deep learning, front-end/back-end interfaces, visualization, and algorithm design. Several members of the team were involved in both patient treatment and model development, as well as research. QA engineers also participated in the FMEA team. All participants had at least 3 years of experience with the RPA. The aim was to identify potential failure modes not captured in previous studies^12^^,^^14^ by exploring whether LLMs could come up with new risks that might be overlooked by human experts. Each team member independently used an LLM of their choice (ChatGPT-4.0, Gemini 2.5 Pro, phi4-reasoning:14B, or the open-source OSS-120B model) to generate potential failure modes. The prompting strategy varied across participants to reflect different approaches to model interaction. Sampling parameters (eg, temperature, max tokens) and output requirements were not standardized across participants, as the goal was to maximize failure mode discovery rather than ensure reproducibility of individual LLM outputs. For example, 1 member used an open-source model to allow greater control over model parameters, another supplemented the LLM with the RPA user manual and workflow guides, whereas others reformulated their prompts to focus on domain-specific risks such as neural-network or data-handling errors. Each user iteratively guided their chosen LLM through multiple queries to elicit and refine failure modes. In addition, each participant instructed their selected LLM to assign scores for occurrence (O), severity (S), and detectability (D) following the 1 to 10 scale definitions in TG-100 Table II.^15^ Scoring prompts were not standardized across participants, though all referenced TG-100 guidelines. RPNs were then calculated for each failure mode by multiplying O, S, and D. Failure modes generated by each participant were pooled and duplicates were consolidated prior to expert review. Where overlapping failure modes had different RPNs, the version with the higher RPN was retained as a conservative approach to avoid underestimating risk. The number of failure modes generated varied by participant depending on the model and prompting strategy used. Failure modes were excluded if classified as "not meaningful" if they were too vague to be actionable or described implausible scenarios not applicable to the RPA workflow (examples: hesitation to raise concerns or ask questions; plan export to incompatible Treatment Planning System (TPS) version). Failure modes were classified as "not relevant" if they pertained to processes outside the RPA workflow (examples: AI inference on unsupported scanner modality like MRI; incorrect combination of models selected). Exclusion decisions were made collectively by the team with consensus required.

Following the initial session, the team reconvened to review all the failure modes identified by the LLMs. The failure modes already identified in prior RPA FMEA were removed from the list. Each potential new failure mode was then reviewed in detail, and RPNs were scored based on consensus across all team members. Although the LLM scores were available, they were not reviewed during the team’s discussions. From this process, failure modes with the highest RPNs were prioritized as the greatest potential risks. These top-ranked failure modes were subsequently shared with 2 major RPA user groups in South Africa for additional feedback, providing the perspective from routine clinical users of the RPA. The study workflow is summarized in Fig. 1.

## Results

### Failure mapping

**Figure 2:**
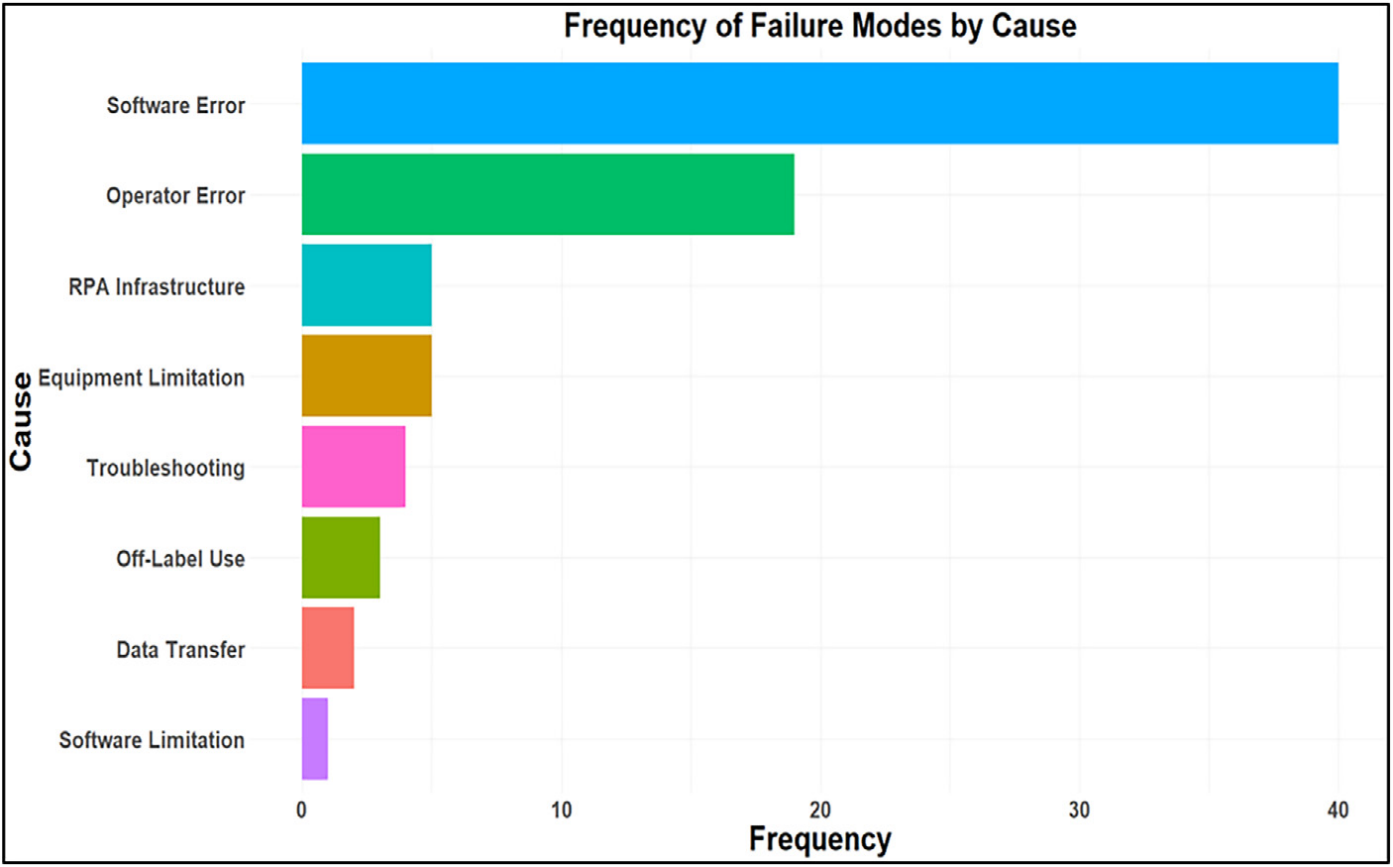
Distribution of identified new failure modes by underlying cause. Bars represent the frequency with which each cause was cited in the Failure Mode and Effects Analysis (FMEA).

**Figure 3:**
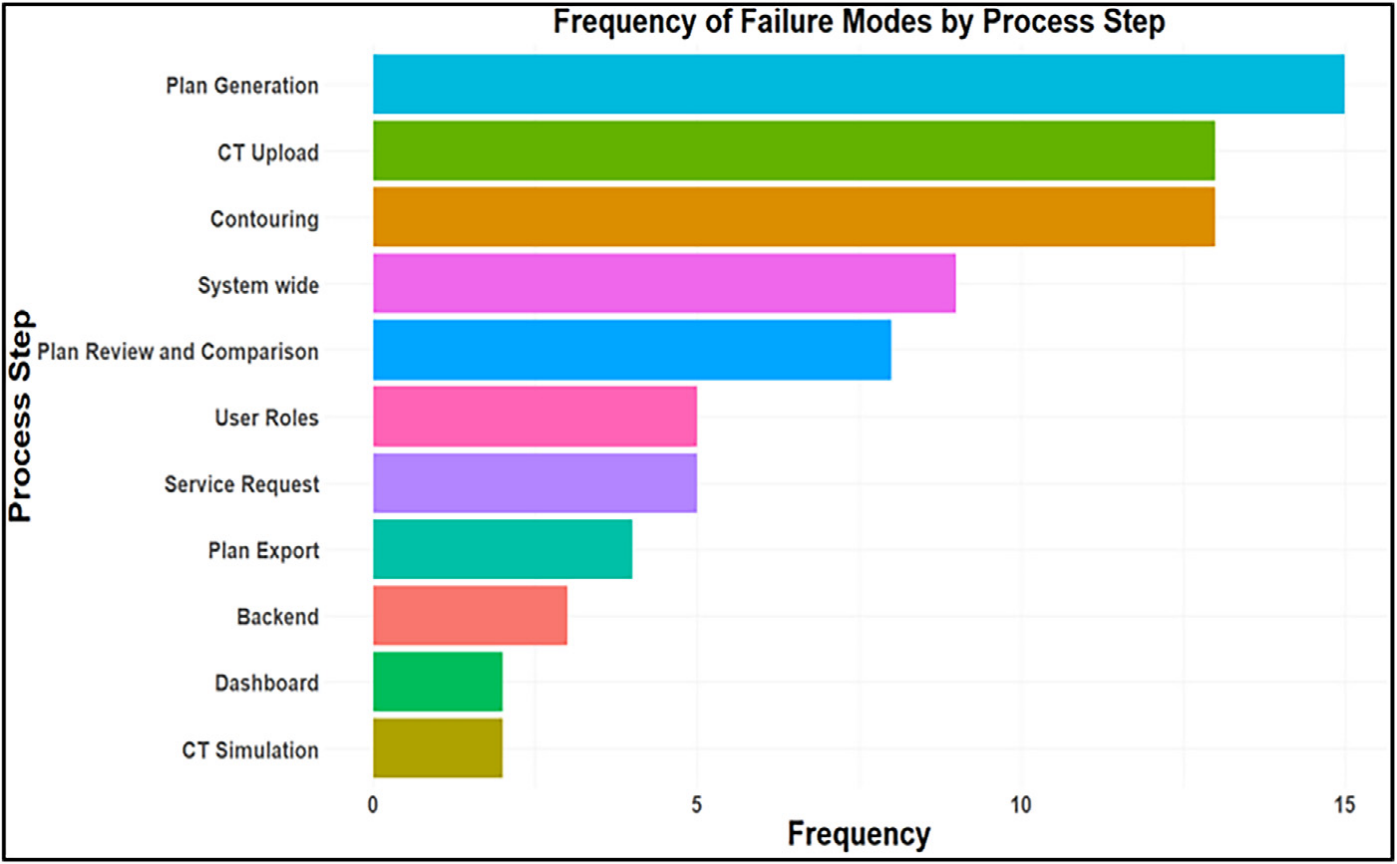
Distribution of identified new failure modes by process step. Bars represent the frequency with which each process step was cited in the Failure Mode and Effects Analysis (FMEA).

The LLMs collectively generated 190 candidate failure modes. Of these, 44 were excluded because they were either not meaningful or not relevant to the RPA workflow, and 67 were identified as duplicates across models. After this review, 79 unique and interpretable failure modes were retained for further analysis. The average RPN was 36 for the new failure modes. All 79 failure modes, along with their O, S, D, and RPN scores (for manual as well as AI), are listed in the Appendix E1. These represent new failure modes not identified in prior RPA FMEA studies; previously identified failure modes were excluded from this list. Each failure mode was classified as one of the underlying causes: software error, operator error, equipment limitations, RPA infrastructure, off-label use, troubleshooting, software limitations, and data transfer. The distribution of error causes is shown in Fig. 2, with software errors accounting for the largest share (n = 40), followed by operator errors (n = 19), and smaller contributions from equipment limitations (n = 5) and RPA infrastructure issues (n = 5). To further understand where failures occur within the clinical workflow, each failure mode was also mapped to a specific process step. The distribution of failure modes by process step is presented in Fig. 3, with the highest number of failures occurring during plan generation (n = 15), CT upload (n = 13), contouring (n = 13), and system-wide issues (n = 9).

### Top failure modes

The top 10 highest scoring failure modes, ranked in descending order of RPN, are presented in Table 1. Among the 79 failure modes, 3 had a risk priority number (RPN) > 125. Notably, all 3 originated from user role-related issues, reflecting potential gaps in accountability and responsibility within the workflow. The failure mode with the highest RPN (rank 1) was “QA step bypassed.” In this scenario, the RPA user assumed that because the system provides automated contouring and planning, all necessary QA checks had already been performed, and no further manual validation was required. However, although the RPA does incorporate some automated QA tools, these are intended to flag possible errors to the user and not to replace manual review. Due to its high severity, moderate likelihood of occurrence, and particularly low detectability, this failure mode was assigned the highest RPN score of 360. Although this might seem similar to the “necessary physics QA not performed” failure mode reported by Nealon et al,^14^ the underlying cause differs. In that earlier study, the issue was linked to automation bias—users over-trusting the automated plan output and omitting manual review. In contrast, the “QA step bypassed” failure identified in this study reflects an operator-driven procedural oversight, where users assume that the software will identify all errors or that QA responsibilities are already covered by the system or another team member. This distinction classifies it as an operator error stemming from role ambiguity rather than a purely automation-related bias, explaining why it did not emerge explicitly in prior expert-driven analyses.

**Table 1:** Top 10 highest scoring new failure modes in the RPA workflow based on expert review

Rank	Failure mode	Process step	Cause	S	O	D	RPN
1	QA step bypassed	User roles	Operator error	8	5	9	360
2	Responsibility silently deferred to others	User roles	Operator error	8	4	8	256
3	No one designated to validate contours	User roles	Operator error	6	4	8	192
4	AI output bias for specific ethnic groups	Contouring	Software Error	5	7	3	105
5	User permissions misconfigured (wrong permissions)	User roles	Off-label use	9	5	2	90
6	Non oncologists accept prescription	User roles	Off-label use	9	5	2	90
7	Software limitation error when planning for complex anatomic variations	Plan generation	Software limitation	8	5	2	80
8	SOP or training content not fully understood	System wide	Operator error	9	8	1	72
9	Plan version mismatch between RPA and local Record & Verify (user imports older draft)	Plan export	Operator error	6	2	6	72
10	Structure misinterpreted due to naming mismatch	Contouring	Operator error	5	2	7	70

*Abbreviations:* AI = artificial intelligence; D = detectability; QA = quality assurance; O = occurrence; RPN = Risk Priority Number; S = severity; SOP = standard operating procedure.

The second and third ranked failure modes were “Responsibility silently deferred to others” (rank 2, RPN = 256) and “No one designated to validate contours” (rank 3, RPN = 192). Both represent potential accountability gaps within the clinical workflow when the RPA is introduced. In the first case, users assume that another team member performs validation, whereas in the second, the task of contour verification is not explicitly assigned to any individual. Together, these errors highlight the risk of oversights being missed when no individual is clearly assigned responsibility. Because contours or plans generated by the RPA may not always be optimal, explicit responsibility for review and validation is essential to prevent unsafe plans from progressing further in the workflow.

### Comparison of manual and AI scoring

**Figure 4:**
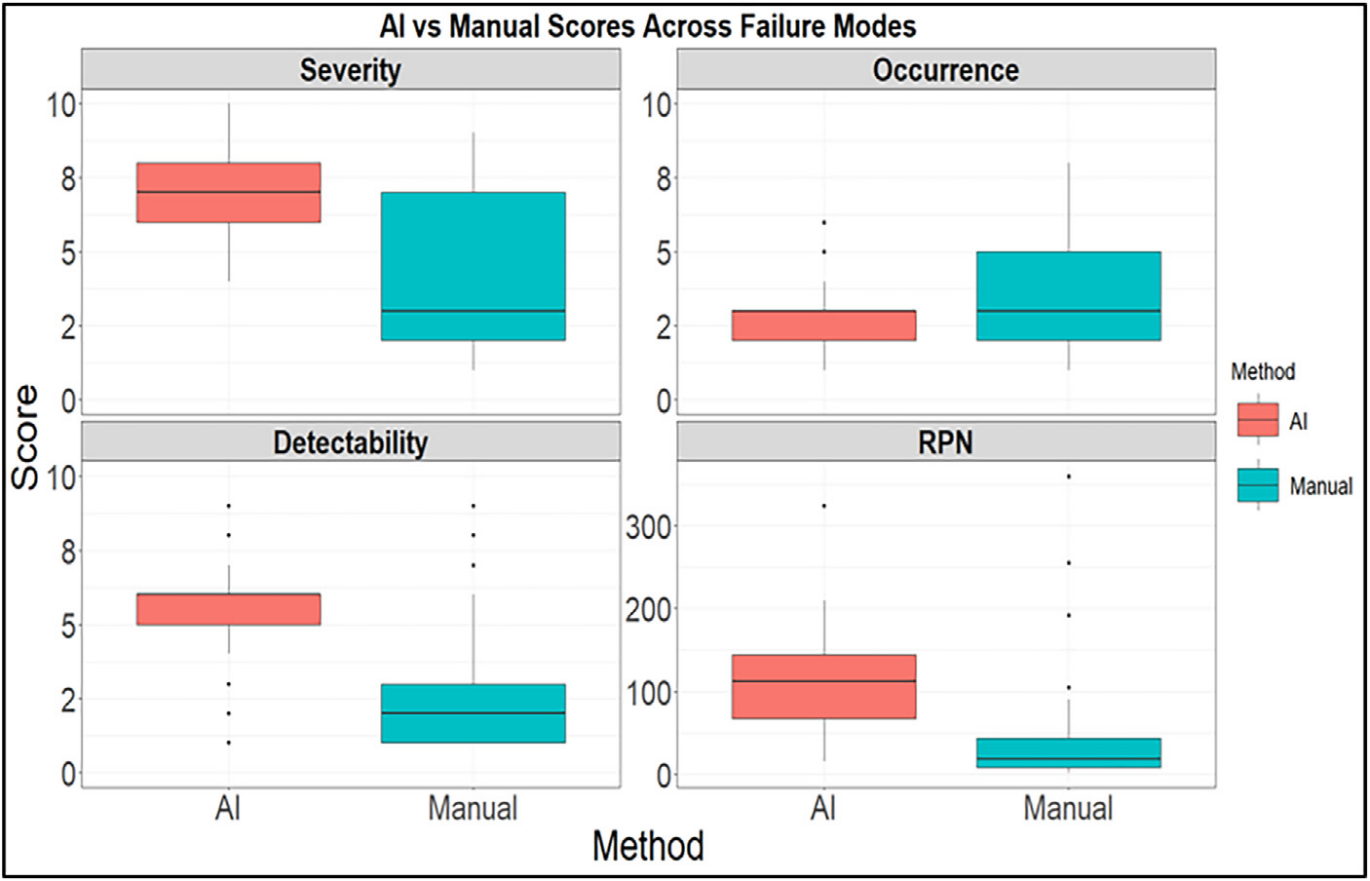
Comparison of manual and artificial intelligence (AI) assigned risk priority number (RPN) scores across 79 new failure modes. Boxplots show the distribution of severity, occurrence, detectability, and RPN values, with AI scoring generally assigning higher severity and detectability values, resulting in higher overall RPNs.

We compared the average risk scores assigned by LLMs to those assigned by the expert team across the 79 new failure modes. On average, LLMs rated severity considerably higher (7.3 vs 4.1) and detectability lower (5.4 vs 2.8), whereas occurrence was similar (2.8 vs 3.3). As a result, the mean RPN assigned by LLMs was more than threefold higher than manual scoring (110 vs 36). Fig. 4 shows the distribution of manual vs AI scores for all 4 metrics. These findings suggest that although LLMs can generate structured scores, their weighting of severity and detectability differs substantially from human experts, underscoring the need for careful review when using AI for quantitative risk prioritization.

### User group review

To contextualize these findings, we shared the highest scoring failure modes (Table 1) with 2 user groups in South Africa and asked them to provide feedback on their likelihood and relevance in routine clinical use. The top failure modes identified in this study were mainly centered on workflow accountability, particularly bypassing manual QA steps and ambiguity in role assignments (ranks 1-3). Both user groups in South Africa acknowledged these as potential risks but mentioned that, in their local practice, there are safety measures in place. For example, medical physicists are responsible for QA and plan checks, with radiation oncologists performing a final review in the TPS, reducing the likelihood that these steps would be missed. However, both groups agreed that the perception that “the RPA has already checked everything” could lead to complacency if clear responsibilities are not properly assigned. Off-label use-related failure modes, such as misconfigured user permissions allowing the wrong person to approve prescriptions, were also discussed (ranks 5-6). Although users agreed that this was theoretically possible, they emphasized that in practice, the RPA has well defined roles and approval rights, making such failures unlikely in their clinical environment, even if user groups make the decision to assign tasks differently. Software-related failure modes received more mixed responses. The users noted challenges in complex cases, particularly in head and neck or postoperative settings, where the RPA may struggle with suboptimal contour or plan quality. They also noted that insufficient local training of the RPA AI models can lead to contouring errors, including occasional bias or cutoffs in body contours (ranks 4, 7). These issues were considered valid but generally detectable during review, with users emphasizing the need for local checks and noting that the system is effective at flagging incompatible inputs. Finally, operator errors caused by, for example, insufficient training on the RPA system or from misunderstanding of structure labels due to local terminology differences (ranks 8-10), were considered low risk in their centers because most staff were proficient in English and familiar with RPA terminology. However, the possibility of accidentally exporting the wrong plan was raised as a practical concern. Overall, this feedback highlights that while some failure modes may be context dependent, the themes identified by LLMs resonated with clinical users, underscoring their potential relevance to broader adoption.

## Discussion

In this study, we demonstrated that LLMs can supplement traditional expert-driven FMEA by identifying new risks within the RPA workflow. The LLMs were able to generate 79 unique failure modes that were not identified in prior analyses, with most of them being software and operational errors. Notably, 3 failure modes exceeded the high-risk threshold (RPN > 125), as reported in a previous study,^14^ and all were tied to role ambiguity and accountability deficiencies (ranks 1-3). These findings suggest that although the RPA provides a robust framework for automated contouring and planning, latent risks remain, particularly at the interface between automation and human oversight. These results show that additional user training should be focused on accountability in order to properly communicate the importance of reviewing the clinical workflows when new tools such as the RPA are introduced into clinical practice. By scoring and reviewing the failure modes identified by LLMs, this study shows that AI can supplement human analysis and make safety assessments more thorough. However, the quality of any FMEA ultimately depends on the expertise and clinical context provided by the panelists, and AI should be viewed as a tool to expand idea generation, not as a replacement for expert judgment.

Our findings build on earlier FMEA studies of the RPA. Kisling et al^12^ carried out the first risk assessment of the 4-field box for cervical cancer using the RPA, with the greatest risks identified coming from automation errors such as incorrect isocenter identification or multileaf collimator or jaw positioning, as well as operator-related issues like inadequate plan review. Nealon et al^14^ expanded the analysis to the full RPA workflow and emphasized the greatest vulnerabilities as inadequate plan review before approval, reliance on the system report as plan documentation, and omission of necessary QA checks, failures that mainly reflected weaknesses in plan preparation, validation, and user accountability. In contrast, our LLM-assisted approach uncovered a broader set of failure modes, including new and more specific examples within categories already recognized in earlier work, as well as additional details in less emphasized areas.

Feedback from the user groups validated the clinical relevance of the newly identified failure modes, even though most had not been encountered in their practice. Their feedback suggests that although local safeguards and role definitions mitigate the likelihood of certain errors, the potential for accountability gaps and overreliance on automation remains credible. This reinforces that the failure modes identified by LLMs, even if context dependent, highlight important areas to monitor as the RPA is adopted more broadly.

This study shows that LLMs can play a useful role in supplementing traditional expert-driven FMEA. Although previous FMEA analyses on the RPA^12^^,^^14^ solely relied on the knowledge and experience of human experts, LLMs were able to broaden the search space and propose failure modes that had not been emphasized before. LLMs highlighted risks related to accountability gaps, infrastructure, and off-label use, which may be overlooked in expert discussions that focus more on familiar operator and automation errors. This shows, LLMs served as a complementary tool that can surface high risks that are less obvious, making the overall safety assessment process more comprehensive.

The relative ordering of failure modes was compared between LLM and expert assigned RPNs to assess whether the 2 approaches prioritized similar risks. The concordance index (C-index) for RPNs was 0.53, indicating only modest agreement in ranking. This suggests that the LLM did not simply assign uniformly higher scores but instead reordered the prioritization of failure modes. Although the LLM tended to be more conservative assigning higher severity and lower detectability values, its identification of which failures posed the greatest overall risk differed meaningfully from expert judgment, underscoring both the subjectivity of manual scoring and the contextual limitations of LLM-based risk estimation. Consistent with the quantitative comparison shown in Fig. 4, the scoring of likelihood, severity, and detectability by LLMs was often vague or inconsistent, requiring expert review to refine rankings and assess clinical relevance. This finding reinforces that, although AI can support risk identification, domain expertise remains indispensable for accurate evaluation and prioritization.

There were a few limitations in this study. The LLM output depended on the quality and detail of the prompts provided. Team members used different models (ChatGPT-4.0, Gemini 2.5 Pro, phi4-reasoning, and OSS-120B) with varying prompting strategies, including supplementing with user manuals or focusing on domain-specific risks. This flexibility was intentional, designed to maximize the discovery of novel failure modes. However, this approach limits our ability to determine whether certain prompting strategies were more effective than others. Future studies with standardized protocols could provide guidance on optimal prompting strategies for AI-assisted FMEA. This work was also limited by feedback from only 2 user groups in South Africa. As the RPA is adopted more widely, future studies should incorporate input from a larger and more diverse set of clinical users to validate the relevance of AI-generated failure modes across different practice settings. Future studies should also evaluate the effectiveness of this LLM-assisted FMEA by implementing mitigations for the highest-risk failure modes and rescoring RPNs, following the approach demonstrated in prior RPA studies.^12^^,^^14^

## Conclusions

In this study, we demonstrated that LLMs can meaningfully supplement traditional expert-driven FMEA of the RPA by identifying new risks that experts may not identify. Feedback from clinical users confirmed that some of these risks are relevant in practice, particularly those related to accountability and software limitations. LLMs therefore serve as a complementary tool that can broaden safety assessments and support safer integration of automation into radiation therapy workflows.

## Disclosures

William Shaw reports financial support was provided by NTeMBI - Nuclear Technologies in Medicine and the Biosciences Initiative. Karl Sachse reports financial support was provided by NTeMBI - Nuclear Technologies in Medicine and the Biosciences Initiative. Laurence Court reports a relationship with Leo Cancer Care that includes: board membership and equity or stocks. Laurence Court reports a relationship with Cancer Prevention and Research Institute of Texas, Fund for Innovation in Cancer Informatics, and Varian Medical Systems that includes: funding grants. Skylar Gay reports financial support was provided by Dr. John J. Kopchick Fellowship. Christoph Trauernicht reports a relationship with National Institutes of Health, National Cancer Institute that includes: funding grants. Christoph Trauernicht reports a relationship with American Association of Physicists in Medicine that includes: speaking and lecture fees. Christoph Trauernicht reports a relationship with South African Association of Physicists in Medicine and Biology that includes: board membership and travel reimbursement. Christoph Trauernicht reports a relationship with International Atomic Energy Agency that includes: travel reimbursement. Christoph Trauernicht reports a relationship with South African Medical Physics Society and Federation of African Medical Physics Organizations that includes: board membership. The other authors declare that they have no known competing financial interests or personal relationships that could have appeared to influence the work reported in this paper.

## Appendix Supplementary materials

Supplementary Image, application 1

## Footnotes

Supplementary material associated with this article can be found in the online version at doi:10.1016/j.adro.2026.102060.
